# Predictors of expressed, felt, and normative needs for informal caregiver counseling: Domestic care for people aged 65+ years

**DOI:** 10.1007/s00391-022-02097-5

**Published:** 2022-08-26

**Authors:** Julia-Sophia Scheuermann, Elmar Gräßel, Anna Pendergrass

**Affiliations:** Center for Health Service Research in Medicine, Department of Psychiatry and Psychotherapy, Uniklinikum Erlangen, Friedrich-Alexander-Universität Erlangen-Nürnberg (FAU), Schwabachanlage 6, 91054 Erlangen, Germany

**Keywords:** Informal caregiver counseling, Needs, Informal caregivers, Home care, Utilization, Angehörigenberatung, Bedürfnisse, Pflegende Angehörige, Häusliche Pflege, Nutzung

## Abstract

**Background:**

Informal caregivers (CGs) often fail to recognize or express a need for informal caregiver counseling (ICC) but ICC is an essential but relatively rarely used support service for CGs.

**Objective:**

Our aim is to identify predictors of CGs’ need for ICC. Stirling et al.’s need model, which includes three needs (expressed, felt, and normative), serves as a theoretical basis.

**Material and methods:**

Analyses are based on cross-sectional data (*n* = 958) from the “Benefits of being a caregiver” study. Predictors of the need to use ICC were analyzed with binary logistic regression. A sensitivity analysis using multiple linear regression was performed for the metric value of normative needs.

**Results:**

We found that 6.8% of CGs currently or have recently used ICC. This expressed need was related to higher education and higher effort in instrumental activities; 24.1% of CGs reported an intention to use ICC in the future. This felt need was related to male gender, lower care level, more problem-focused coping, and a desire for more informal help. Objective need for ICC (normative need), which was related to a higher burden of care, less experienced benefits, and negative relationship quality, was reported by 21.4% of CGs. According to a sensitivity analysis, higher education, a desire for informal help, and living in separate households also predicted a normative need for counseling.

**Discussion:**

Current utilization is significantly lower than the subjectively perceived and objectively existing need for ICC. The identified predictors provide initial strategies for motivating more CGs to use ICC.

**Supplementary Information:**

The online version of this article (10.1007/s00391-022-02097-5) contains supplementary material, which is available to authorized users.

## Abstract (de)

**Hintergrund:**

Pflegende Angehörige (pA) erkennen oder äußern oft nicht den Beratungsbedarf. Dabei ist Angehörigenberatung (AB) ein essenzielles, jedoch relativ selten genutztes Unterstützungsangebot für pA.

**Fragestellung:**

Ziel ist es, Prädiktoren für den Bedarf von AB bei den pA zu ermitteln. Als theoretische Grundlage dient das Need-Modell von Stirling et al., das drei Bedürfnisse („expressed“, „felt“ und „normative“) umfasst.

**Material und Methoden:**

Die Analysen basieren auf Querschnittsdaten (*n* = 958) aus der Studie „Benefits of Being a Caregiver“. Prädiktoren für die Bedürfnisse zur Nutzung der AB wurden mit binär logistischen Regressionen analysiert. Für die metrische Ausprägung von „normative need“ wurde zusätzlich eine Sensitivitätsanalyse mittels multipler linearer Regression durchgeführt.

**Ergebnisse:**

AB wird derzeit oder in letzter Zeit von 6,8 % der pA genutzt. Dieser „expressed need“ steht in Zusammenhang mit höherer Bildung und höherem Aufwand für instrumentelle Aktivitäten; 24,1 % der pA gaben an, dass sie beabsichtigen, AB in Zukunft zu nutzen. Dieser „felt need“ stand in Zusammenhang mit dem männlichen Geschlecht, niedrigerem Pflegegrad, problemfokussierterem Coping und dem Wunsch nach mehr informeller Hilfe. Der objektive Bedarf an AB („normative need“), der mit einer höheren Pflegebelastung, weniger erlebten Benefits und einer negativen Beziehungsqualität zusammenhing, wurde von 21,4 % der pA angegeben. Gemäß Sensitivitätsanalyse sind zusätzlich höhere Bildung, Wunsch nach informeller Hilfe und Leben in getrennten Haushalten prädiktiv für den objektiven Beratungsbedarf.

**Diskussion:**

Die tatsächliche Inanspruchnahme ist deutlich geringer als der subjektiv empfundene und objektiv vorliegende Bedarf an AB. Die ermittelten Prädiktoren liefern erste Ansatzpunkte, um mehr pA zu motivieren, AB zu nutzen.

Informal caregivers (CGs) are often stressed from caring for a long-term care-dependent older person and a corresponding lack of time [29]. Therefore, CGs express the need for formal support and counseling [1]. In Germany, informal caregiver counseling (ICC) is one of many support services that CGs can receive while giving care [11]. To better tailor such support to CGs’ needs, we aim to identify factors associated with the use of ICC and link them to CGs’ needs in line with Stirling et al.’s [27] need model.

## Background

### Informal caregiver counseling (ICC)

In 2009, CGs won the right to professional and free ICC in Germany. CGs need information on many topics [24] and need to know how to give care and how to find support to help them balance their caregiving role with their own needs [3]. Offers of support (e.g., ICC) are necessary to give CGs some relief [23]. ICC is a psychoeducational approach for improving CGs’ abilities to cope with informal care [2, 11]. Through ICC, CGs can discuss their situation and get practical advice and information on how to get support. Thus, ICC guides CGs through the care process and helps them get further support. Despite CGs reported need for more help [27] and the potential of ICC to help CGs, CGs rarely use ICC [12].

### Need concept

CGs’ needs and burdens are linked to their use of services [14]. Although CGs expect their needs to be addressed, counselors often fail to do so [16, 27]. It is unclear whether CGs are not expressing their needs adequately enough or whether CGs are unable to even perceive their own needs because they feel obligated to provide care [20]. Therefore, it is necessary to identify the predictors of CGs’ needs in order to meet them, to adapt services accordingly, and lessen the burden on CGs [4].

**Fig. 1:**
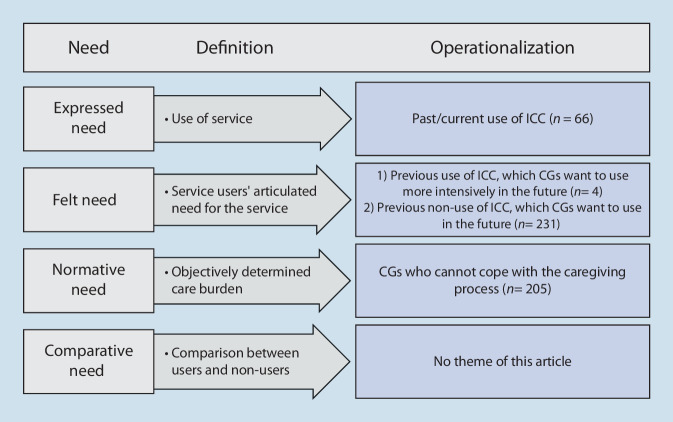
Operationalization of Stirling et al.’s [27] need model, modified for the present study

The typology of needs [27] differentiates between expressed, felt, normative, and comparative needs. Expressed need indicates the actual use of support services. Felt need includes the desire to use these services. Normative need refers to the objectively apparent need for support services, and comparative need compares users with non-users. Fig. 1 presents the theoretical model in terms of the needs considered in the present study. Initial research results show that care receivers’ (CRs) cognitive performance level [14] and CGs’ age [13], as well as problem-focused coping [10] predict ICC use. Problem-focused coping involves people orienting themselves to the situation and its framework conditions [10]. Therefore, persons with this coping style seek external support in coping with the situation. For nondemented CRs, CGs’ employment also predicts ICC use [13]. However, the relationship between individual needs and ICC use has yet to be clarified. Thus, our research question is: “Which factors are significant predictors of CGs’ expressed, felt, and normative needs for ICC use?”

## Method

### Study design and participants

Data were collected in the “Benefits of being a caregiver” study. Between October 2019 and March 2020, 50 care assessors from the Medical Service of the Bavarian Health Insurance (MD) distributed 5000 self-report questionnaires to statutorily insured informal CGs who applied for an initial grade or an increase in CRs’ care level at the MD. By returning the completed questionnaire, 1082 CGs (21.64%) provided informed consent.

The final sample included 958 cases after 124 cases were excluded because they were missing information on ICC use (*n* = 3) or the CR’s age was under 65 years (*n* = 121). Sample characteristics are given in supplementary material 1.

### Instruments

The questionnaire contained various scales related to the use of support services, the care situation, CGs’ state of mind (coping, benefits, subjective burden), and CGs’ and CRs’ demographic information. In this study, we focused in particular on variables that can be influenced in order to derive improvements for CGs’ situation. The focus was primarily on constructs, such as relationship quality, that have received little attention in the ICC to date.

#### Outcome variables

The study included three different outcome variables: expressed, felt, and normative needs for ICC use. Thus, with ICC, we focused on one support service from the Resource Utilization in Dementia (RUD) questionnaire [28]. Expressed and felt needs were evaluated dichotomous by asking: a) whether the CGs currently use or recently used ICC (expressed need); b) whether the CGs already use and would like to use ICC more intensively in the future (felt need I); and whether the CGs have never used ICC but would like to use it in the future (felt need II). Normative need was operationalized, analogous to Stirling et al. [27], through CGs’ subjective perception of their ability to cope with care, assessed with the question “How do you currently assess your ability to cope with caregiving?” The item was rated on a 10-point scale ranging from 0 (completely succeeding) to 9 (not succeeding at all) and subsequently dichotomized at a score of 4 for better comparability of the needs.

#### Independent variables

Table 1 provides a brief overview of the independent variables considered. Supplementary material 2 contains detailed descriptions of the variables and instruments.

**Table 1:** Independent variables

Variable	Instrument or item
Subjective care burden	Burden Scale for Family Caregivers, short version
Benefits	Benefits of Being a Caregiver Scale
Coping behavior	6 items from Brief COPE
Coping with care	“How do you currently assess your ability to cope with caregiving?”
Home care motivation	“What is the main reason that you are administering care at home?”
Relationship quality actual	“How do you rate the quality of the relationship between you and the person you support or care for?”
Relationship quality before caregiving	“How would you rate the quality of the relationship between you and the person you support or care for before they needed your help or support?”
Informal help from relatives/friends	“Are relatives or friends currently helping you administer care?”
Wish for more informal help from relatives/friends	“Would you like relatives or friends to help more with care?”
Informal care time	Activities of daily living, instrumental activities of daily living, supervision
Sociodemographic characteristics	CG: age, gender, employment, education, relationship, living situation, care duration CR: age, gender, cause of care dependency, care level

*CG* caregiver, *CR* care receiver

### Statistical analyses

Statistical analyses were calculated with IBM SPSS version 28 for Windows (IBM Corporation, Armonk, NY, USA). The cross-sectional baseline data were included in the analyses. The three needs were the dichotomous dependent variables. Categorical predictors with more than two values were recoded into dichotomous values.

Using bivariate analyses (t-tests for metric and χ^2^-tests for nominal variables), we wanted to determine whether the three needs were related to any of the other variables.

Because the dependent variables were dichotomous, we used binary logistic regression to identify possible predictors of the three needs in the context of ICC use. The logistic regression analyses were computed in blocks. To control for CGs’ age, gender, and education, we used the enter method in the first block. Due to the large sample, all other non-multicollinear predictors were added via forward selection in the second block. If predictors exhibited multicollinearity (Pearson *r* > 0.60), the predictor with a higher bivariate correlation with the outcome was included in the regression model. The thresholds were *p* = 0.01 for inclusion and* p* = 0.10 for removal of a predictor. Due to the artificial dichotomization of normative need, a sensitivity analysis was conducted with multiple linear regression.

## Results

### Descriptive

Descriptive data, sample characteristics, and statistical results for the three needs are presented in supplementary material 1. Multicollinearity analyses showed high intercorrelations between employment status and CGs’ age for all three needs. Due to the lower bivariate correlation of employment with the needs, this variable was not included in the regression analyses.

### Expressed need

Seven percent of the CGs reported using ICC currently or recently. The binary logistic regression analysis (Table 2) resulted in a significant model (χ^2^ = 14.71 (df:4),* p* = 0.005) with two significant predictors. More years of education and higher Instrumental Activities of Daily Living (IADL) effort predicted ICC use. Nagelkerke’s *R*^2^ was 0.04; therefore, the examined variables explained 4% of the variance in ICC use.

**Table 2:** Binary logistic regression for the three needs as dependent variables; model: enter (Block I), forward selection (Block II)

Predictor	Expressed need				Felt need				Normative need			
	*B*	*P*^a^	OR	95% CI (OR)	*B*	*P*^a^	OR	95% CI (OR)	*B*	*P*^a^	OR	95% CI (OR)
*Block I*^b^												
Gender^c^	−0.29	0.391	0.75	[0.39;1.45]	0.65	**<** **0.001**	1.91	[1.34;2.69]	0.01	0.980	1.01	[0.66;1.53]
Age	−0.01	0.231	0.99	[0.97;1.01]	−0.01	0.079	0.99	[0.98;1.00]	−0.01	0.517	0.99	[0.98;1.01]
Education	0.09	**0.034**	1.09	[1.01;1.19]	−0.03	0.318	0.97	[0.92;1.03]	−0.06	0.085	0.95	[0.89;1.01]
*Block II*^d^												
IADL (h/day)	0.14	**0.002**	1.15	[1.05;1.26]	–	–	–	–	–	–	–	–
Care level	–	**–**	–	–	−0.16	**0.006**	0.85	[0.76;0.95]	–	–	–	–
Problem-focused coping^e^	–	–	–	–	0.11	**0.006**	1.12	[1.03;1.21]	–	–	–	–
Desire for informal help (yes)^f^	–	–	–	–	0.47	**0.004**	1.60	[1.16;2.20]	–	–	–	–
Subjective care burden^g^	–	–	–	–	–	–	–	–	−0.10	**<** **0.001**	0.90	[0.88;0.93]
Benefits^h^	–	–	–	–	–	–	–	–	0.02	**0.005**	1.02	[1.01;1.04]
Actual relationship quality (positive)	–	–	–	–	–	–	–	–	0.69	**<** **0.001**	1.99	[1.40;2.82]

*N* = 958, *B* non-standardized regression coefficient *B, CI* confidence interval, *OR* odds ratio; expressed and felt need: 0 = no need, 1 = need; normative need: 0 = need, 1 = no need

^a^ *p* < 0.05 printed in bold letters

^b^ Adjustment variables relate only to caregivers

^c^ Dichotomous variable: female = 0, male = 1

^d^ Final regression model—expressed need: Nagelkerke’s *R*^2^ = 0.039, χ^2^ = 14.709, *p* = 0.005, 1 step; felt need: Nagelkerke’s *R*^2^ = 0.059, χ^2^ = 38.596, *p* < 0.001, 3 steps; normative need: Nagelkerke’s *R*^2^ = 0.190, χ^2^ = 125.708, *p* < 0.001, 3 steps

^e^ Measured with 2 Items from the Brief COPE, range 0–8

^f^ Dichotomous variable: Caregiver would like more help with caregiving from friends or family

^g^ Measured with the Burden Scale for Family Caregivers—short, range 0–30

^h^ Measured with the Benefits of Being a Caregiver Scale, range 0–56

### Felt need

Because only four cases were available for felt need I, the analysis refers to felt need II. About 24.1% of the CGs articulated a future need for ICC (*n* = 231). The binary logistic regression analysis (Table 2) resulted in a significant model (χ^2^ = 38.59 (df:6), *p* < 0.001) with four significant predictors. Male gender, lower care level, more problem-focused coping, and a desire for more informal help predicted future ICC use if they had not used it before. Nagelkerke’s *R*^2^ was 0.06; therefore, the examined variables explained 6% of the variance in future ICC use.

### Normative need

About 21.4% of the CGs could not cope successfully with caregiving and were in need of ICC. The binary logistic regression analysis (Table 2) resulted in a significant model (χ^2^ = 125.71 (df:6), *p* < 0.001) with three significant predictors. Higher subjective care burden, less received benefits, and lower current relationship quality predicted not coping successfully with care and showing a normative need for ICC. Nagelkerke’s *R*^2^ was 0.19; therefore, the examined variables explained 19% of the variance in a normative need for ICC.

The sensitivity analysis (supplementary material 3) shows that in addition to the predictors we mentioned the variables higher education (*p* = 0.020), desire for more informal help (*p* < 0.001), and no co-residence (*p* < 0.001) were significant.

## Discussion

The aim of the analysis was to identify predictors of CGs’ expressed, felt, and normative needs to use ICC. In general, the data showed a gap between low use of ICC on the one hand and a higher subjective and objective need for ICC on the other. The low use of ICC is consistent with the literature [9, 15, 25, 26]. A possible explanation is that the number of ICC users is higher than reported, as some CGs did not perceive the counseling they received as such and therefore did not report it in the questionnaire. Lack of overview and knowledge about support services and their availability are cited as other main reasons for not using support services [5]. The results also show that the majority of CGs do not have the needs assumed by professionals. Since CGs are often unaware of their own needs [6], these cannot be met within the ICC setting. This is based on a top-down process in which care counselors seek to address potentially important issues. However, due to CGs’ unmet needs, it becomes apparent that a bottom-up procedure according to CGs’ main topics would be more helpful. This will require more intensive training of care counselors.

### Expressed need

In this study, education and IADL were significant predictors of current or recent ICC use. In line with the literature [9, 17, 18], a high level of education had a strong influence on the use of support services. CGs with higher education use different information-seeking and source-utilization strategies to obtain information. By taking advantage of ICC, these CGs may expect to find strategies to better balance work and caregiving. They may also have more work-related opportunities to take advantage of such services. Because of the relationship between education and socioeconomic status, these CGs have more financial resources to use support services in the caregiving situation. In contrast to the literature, in our study there was a correlation between higher IADL effort and expressed need [7]. However, the overall time spent on caregiving and activities of daily living (ADL) are considered predictors of professional help seeking [13, 19]. Furthermore, CGs show a need for information and support regarding IADL [20]. For ADL tasks, CGs can seek relief from care services. For IADL tasks, however, such possibilities do not exist in the German care system. To cope with the organization of these tasks, CGs therefore still lack support services that relieve them of IADLs. This means that mostly CGs with a high level of education and high objective burden have been reached with ICC so far.

### Felt need

For CGs who had experienced ICC, results showed that they did not want more ICC. For previous non-users of ICC with the intention to use ICC in the future, the study showed the following predictive factors: male gender, desire for more informal help, more problem-focused coping, and lower care level. In line with the literature [18, 22], we found that men were more likely to use ICC in future and ask for support in the care process. This could be interacting with CGs’ employment. Men who are more often employed and less often CGs, seek outside support to better balance work and caregiving. In contrast, women are still exposed to the role stigma of shouldering the main burden of caregiving. In this respect, they are more likely to give up work and often take on more time-intensive care than men. Therefore, men are more likely to make use of support services, even though they are less likely to be CGs in terms of absolute frequency. In addition, the desire for more informal help contributed significantly to the intention to use ICC. Lack of family support and available informal resources predict ICC use [13]. This finding is in line with our finding that problem-focused coping strategies increased the likelihood of using ICC. These strategies involve actively seeking external help and thus help CGs cope with stress in the care process [10, 21]. A lower level of care also contributed to a higher probability that CGs will use ICC.

### Normative need

Predictors of a normative need for ICC because CGs could not cope with the current situation were high subjective care burden, lower benefits, current negative relationship quality, higher level of education, co-residence, and a desire for more informal help. In line with Lüdecke et al. [18], we found that subjective care burden predicted a normative need for ICC use. The co-residence of CG and CR proves to be a possible predictor of normative need. Due to the association found in the literature between co-residence and subjective care burden [8], there is a need to determine whether co-residence is a mediator or predictor of normative needs. A current positive quality of the relationship between the CG and CR and experienced benefits reduced the normative need for ICC and went along with better coping with care. The predictors we identified show that closeness, relationship quality, and subjective burden (i.e., psychological components) dominate the relationship with normative need.

## Strengths and limitations

The study has several strengths: a heterogeneous sample with different durations and degrees of care, the analysis of nearly 1000 questionnaires representative of legally insured CGs in Bavaria who applied for a care level or upgrade. But there are also some limitations. First, although a brief description of ICC was available in the questionnaire, there was no detailed specification of ICC. Therefore, we cannot be sure that all CGs understood ICC to mean the same thing or that all types of ICC were included in the definition. Second, due to the subjective assessment of current or recent ICC use, no precise definition of duration of use was possible. Third, the available data were cross-sectional, and thus, do not allow causal conclusions. Fourth, no data were collected on health, which may contribute to the felt needs in particular [27].

## Conclusion

- CGs’ need for ICC exists, but only 7% of CGs have used it. CGs’ education predicts ICC use. Further research should address the question of how to reach all CGs, especially those with lower education levels.
- Many non-users of ICC have the desire to use it. The barriers to use we found (female gender, lower care level, low problem-focused coping, and no desire for informal help) should be considered in providing ICC.
- One out of five CGs feel like they cannot cope with the care situation, but most of them do not seek help. Caregiver counselors and policy makers should focus on closing this gap.

## Supplementary Information

Supplement material 1: Table T1: Sample characteristics (*N* = 958)

Supplement material 2: Description of independent variables

Supplement material 3: Table T3: Sensitivity analysis for normative need; model: enter (Block I), forward selection (Block II)

## Abbreviations

ADL: Activities of daily living
Benefits: Benefits to the caregiver from giving care at home
CGs: Informal caregivers
CRs: Care receivers
IADL: Instrumental activities of daily living
ICC: Informal caregiver counseling
MD: Medical Service of the Bavarian Health Insurance
RUD: Resource utilization in dementia

## Declarations

All described studies in humans were conducted with the approval of the responsible ethics committee, in accordance with national law and the Declaration of Helsinki of 1975 (in the current, revised version). Informed consent was obtained from all participants. Approval for the study and the manner in which consent was obtained was granted by the Ethics Committee of the Medical Faculty of the University of Erlangen (Registration Number 220_20 B).
