# Supplementary material for: Predictors of expressed, felt, and normative needs for informal caregiver counseling: Domestic care for people aged 65+ years
Source: Z Gerontol Geriatr. 2022 Aug 26;56(5):395–401. doi: 10.1007/s00391-022-02097-5 (PMC10406654; doi:10.1007/s00391-022-02097-5)
Supplement: Supplementary file 1 — Supplement material 1: Table T1: Sample characteristics (N = 958) [file 391_2022_2097_MOESM1_ESM.docx]

**Supplement material 1**

Table T1: Sample characteristics (*N*=958)

|  |  | *Expressed need* | | | *Felt need II* | | | | *Normative need* | | |
| --- | --- | --- | --- | --- | --- | --- | --- | --- | --- | --- | --- |
| Variables | Cohort  *M*(*SD*) or *n*(%) | Yes *M*(*SD*) or *n*(%)  *n*=66 (6.9) | No *M*(*SD*) or *n*(%)  *n*=892 (93.1) | *p ^a,b^* | Yes *M*(*SD*) or *n*(%)  *n*=231 (24.1) | No *M*(*SD*) or *n*(%)  *n*=727 (75.9) | *p ^a,b^* | Yes *M*(*SD*) or *n*(%)  *n*=205 (21.4) | | No *M*(*SD*) or *n*(%)  *n*=753 (78.6) | *p ^a,b^* |
| ***Caregiver*** |  |  |  |  |  |  |  |  | |  |  |
| Age (years) | 62.07 (12.63) | 60.18 (12.82) | 62.21 (12.62) | .209^a^ | 61.10 (12.73) | 62.37 (12.60) | .183^a^ | 61.81 (12.69) | | 62.14 (12.63) | .742^a^ |
| Gender (male) | 234 (24.4) | 13 (19.7) | 221 (24.8) | .458^b^ | 73 (31.6) | 161 (22.1) | .005^b^ | 42 (20.5) | | 192 (25.5) | .144^b^ |
| Employment (yes) | 459 (47.9) | 32 (48.5) | 427 (47.9) | 1.00^b^ | 116 (50.2) | 343 (47.2) | .450^b^ | 99 (48.3) | | 360 (47.8) | .937^b^ |
| Education (years) | 10.67 (2.68) | 11.32 (3.36) | 10.62 (2.61) | .105^a^ | 10.61 (2.61) | 10.69 (2.70) | .708^a^ | 10.81 (2.70) | | 10.64 (2.67) | .424^a^ |
| Relationship (spouses, yes) | 291 (30.4) | 24 (36.4) | 267 (29.9) | .270^b^ | 68 (29.4) | 223 (30.7) | .743^b^ | 56 (27.3) | | 235 (31.2) | .305^b^ |
| Subjective care burden (BSFC-s) | 16.73 (7.48) | 18.38 (7.62) | 16.61 (7.45) | .063^a^ | 17.62 (7.00) | 16.45 (7.60) | .038^a^ | 21.02 (6.34) | | 15.56 (7.34) | <.001^a^ |
| Benefits (BBCS) | 26.10 (12.34) | 27.53 (13.63) | 25.99 (12.24) | .328^a^ | 27.17 (11.64) | 25.76 (12.54) | .130^a^ | 22.93(11.25) | | 26.96 (12.49) | <.001^a^ |
| Avoidant coping | 6.15 (1.56) | 6.38 (1.73) | 6.13 (1.55) | .214^a^ | 6.02 (1.61) | 6.19 (1.53) | .157^a^ | 5.98 (1.56) | | 6.19 (1.56) | .082^a^ |
| Emotion-focused coping | 3.72 (2.22) | 4.21 (2.40) | 3.68 (2.21) | .061^a^ | 3.83 (2.16) | 3.68 (2.24) | .389^a^ | 3.75 (2.15) | | 3.71 (2.24) | .805^a^ |
| Problem-focused coping | 4.04 (1.97) | 4.21 (2.14) | 4.03 (1.96) | .461^a^ | 4.37 (1.87) | 3.94 (1.99) | .004^a^ | 4.28 (1.96) | | 3.97 (1.97) | .046^a^ |
| Self-determined care motivation (yes) | 235 (24.5) | 12 (18.2) | 223 (25.0) | .238^b^ | 62 (26.8) | 173 (23.8) | .380^b^ | 57 (27.8) | | 178 (23.6) | .234^b^ |
| Relationship quality actual (positive) | 551 (57.5) | 39 (59.1) | 512 (57.4) | .897^b^ | 120 (51.9) | 431 (59.3) | .056^b^ | 73 (35.6) | | 478 (63.5) | <.001^b^ |
| Relationship quality before caregiving (positive) | 568 (59.3) | 35 (53.0) | 533 (59.8) | .301^b^ | 123 (53.2) | 445 (61.2) | .038^b^ | 104 (50.7) | | 464 (61.6) | .006^b^ |
| ***Care receiver*** |  |  |  |  |  |  |  |  | |  |  |
| Age (years) | 82.13 (7.05) | 81.06 (7.10) | 82.21 (7.04) | .201^a^ | 82.08 (6.80) | 82.15 (7.12) | .898^a^ | 82.50 (6.68) | | 82.03 (7.14) | .403^a^ |
| Gender (male) | 316 (33.0) | 23 (34.8) | 293 (32.8) | .786^b^ | 70 (30.3) | 246 (33.8) | .336^b^ | 65 (31.7) | | 251 (33.3) | .676^b^ |
| Dementia (yes) | 118 (12.3) | 12 (18.2) | 106 (11.9) | .171^b^ | 29 (12.6) | 89 (12.2) | .909^b^ | 30 (14.6) | | 88 (11.7) | .280^b^ |
| Level of care | 1.93 (1.32) | 2.32 (1.21) | 1.90 (1.32) | .014^a^ | 1.74 (1.31) | 1.99 (1.32) | .013^a^ | 1.86 (1.40) | | 1.95 (1.30) | .389^a^ |
| ***Care situation*** |  |  |  |  |  |  |  |  | |  |  |
| Coping with care | 3.08 (2.02) | 2.95 (1.76) | 3.09 (2.04) | .604^a^ | 3.31 (2.02) | 3.01 (2.02) | .049^a^ | - | | - | - |
| Coresidence (yes) | 505 (52.7) | 38 (57.6) | 467 (52.4) | .445^b^ | 116 (50.2) | 389 (53.5) | .406^b^ | 96 (46.8) | | 409 (54.3) | .059^b^ |
| Care duration (months) | 48.34 (78.59) | 49.82 (61.01) | 48.23 (79.76) | .874^a^ | 51.65 (102.94) | 47.29 (69.13) | .463^a^ | 52.53 (73.61) | | 47.20 (79.89) | .389^a^ |
| Care of several people (yes) | 59 (6.2) | 4 (6.1) | 55 (6.2) | 1.00^b^ | 17 (7.4) | 42 (5.8) | .432^b^ | 13 (6.3) | | 46 (6.1) | .871^b^ |
| ADL (h/d) | 2.70 (2.24) | 3.15 (2.59) | 2.66 (2.21) | .145^a^ | 2.45 (2.05) | 2.77 (2.30) | .058^a^ | 2.71 (2.19) | | 2.69 (2.26) | .927^a^ |
| IADL (h/d) | 3.45 (2.26) | 4.27 (3.01) | 3.39 (2.18) | .023^a^ | 3.55 (2.20) | 3.43 (2.27) | .477^a^ | 3.53 (2.32) | | 3.43 (2.24) | .581^a^ |
| Supervision (h/d) | 2.70 (3.25) | 3.51 (3.42) | 2.64 (3.23) | .038^a^ | 2.43 (3.11) | 2.79 (3.29) | .149^a^ | 3.35 (3.57) | | 2.52 (3.14) | .003^a^ |
| Informal help received (yes) | 576 (60.1) | 41 (62.1) | 535 (60.0) | .795^b^ | 149 (64.5) | 427 (58.7) | .124^b^ | 125 (61.0) | | 451 (59.9) | .810^b^ |
| Informal help desired (yes) | 582 (60.8) | 43 (65.2) | 539 (60.4) | .514^b^ | 159 (68.8) | 423 (58.2) | .004^b^ | 159 (77.6) | | 423 (56.2) | <.001^b^ |

*Note*. *M*=Mean; *SD*=Standard deviation. Subjective care burden measured with the BSFC-s, range 0 – 30; Benefits measured with the BBCS, range 0 – 56; avoidant, emotion-focused, problem-focused coping: measured with two items each subscale from the Brief COPE, range 0 – 8; Dementia = Cause of care is dementia; Level of care, range 0 – 5; Coping with care, range 0 – 9; ADL = Activities of daily living; IADL = Instrumental activities of daily living.

^a^t-test for metric variables.

^b^Fisher’s Exact test for dichotomous variables.
