# Supplementary material for: Predictors of expressed, felt, and normative needs for informal caregiver counseling: Domestic care for people aged 65+ years
Source: Z Gerontol Geriatr. 2022 Aug 26;56(5):395–401. doi: 10.1007/s00391-022-02097-5 (PMC10406654; doi:10.1007/s00391-022-02097-5)
Supplement: Supplementary file 2 — Supplement material 2: Description of independent variables [file 391_2022_2097_MOESM2_ESM.docx]

**Supplement material 2 – Description of independent variables**

CGs’ subjective burden was measured with the 10-item short version of the Burden Scale for Family Caregivers (BSFC-s) [2] on a 4-point scale ranging from 0 (not true) to 3 (true exactly). The total score ranges from 0 to 30 such that the higher the score, the higher the CG’s subjective burden.

CGs´ benefits from informal caregiving were assessed with the newly developed 14-item Benefits of Being a Caregiver Scale (BBCS), answered on a 5-point scale ranging from 0 (strongly disagree) to 4 (strongly agree). The total score ranges from 0 to 56 such that the higher the score, the higher the benefits to the CG.

CGs´ general coping behavior was measured with six items from the Brief COPE questionnaire [1]. It includes two items of each of three scales (i.e., problem-focused, emotion-focused, and avoidant coping) that were evaluated on a 5-point scale ranging from 0 (strongly disagree) to 4 (strongly agree).

CGs´ assessment of their subjective perception of their ability to cope with the specific care situation was recorded with a 10-point scale ranging from 0 (completely succeeding) to 9 (not succeeding at all).

Home care motivation was evaluated by stating the reason for giving care at home. CGs could choose from seven response alternatives [3], which were dichotomized into self-determined (CGs’ desire) and non-self-determined (other care motives; e.g., sense of commitment, care place too expensive, no care place found).

The quality of the relationship between CG and CR was assessed currently and before the need for care, using a three-level pictorial response format with the qualities negative, neutral, and positive. Because we assumed that social desirability was present, this response format was subsequently dichotomized into positive and non-positive (which included neutral and negative) relationship quality.

The three aspects of informal care time – Activities of Daily Living (ADL), Instrumental Activities of Daily Living (IADL), and supervision – were each evaluated with one item. For each of the three aspects, the average daily number of hours spent giving care was also measured.

Furthermore, the desire for current and future informal help for care was assessed with the two items “Are relatives or friends currently helping you administer care?” and “Would you like relatives or friends to help more with care?”

We also assessed sociodemographic and background characteristics (e.g., CGs´ and CRs´ age and gender), CGs´ employment and educational attainment, relationship (dichotomized into spouses or non-spouses), living situation, duration of care in months, cause of care dependency, and CRs´ care level.

## References

1. Carver CS (1997) You want to measure coping but your protocol's too long: consider the brief COPE. Int J Behav Med 4:92-100

2. Graessel E, Berth H, Lichte T et al. (2014) Subjective caregiver burden: validity of the 10-item short version of the Burden Scale for Family Caregivers BSFC-s. BMC Geriatr 14:1-9

3. Gräßel E (2000) Warum pflegen Angehörige? Ein Pflegemodell für die häusliche Pflege im höheren Lebensalter. GeroPsych 13:85-94
