# Supplementary material for: Predictors of expressed, felt, and normative needs for informal caregiver counseling: Domestic care for people aged 65+ years
Source: Z Gerontol Geriatr. 2022 Aug 26;56(5):395–401. doi: 10.1007/s00391-022-02097-5 (PMC10406654; doi:10.1007/s00391-022-02097-5)
Supplement: Supplementary file 3 — Supplement material 3: Table T3: Sensitivity analysis for normative need; model: enter (Block I), forward selection (Block II) [file 391_2022_2097_MOESM3_ESM.docx]

**Supplement material 3**

Table T3 – Sensitivity analysis for normative need; model: enter (Block I), forward selection (Block II)

| Predictor | Regression coefficient *B^a^* | *P^b^* | 95% Confidence interval |
| --- | --- | --- | --- |
| Block I^c^ |  |  |  |
| Gender^d^ | .08 | .572 | [-0.19;0.35] |
| Age | .01 | .572 | [-0.01;0.01] |
| Education (years) | .05 | **.020** | [0.01;0.10] |
| Block II^e^ |  |  |  |
| Subjective care burden^f^ | .08 | **<.001** | [0.06;0.10] |
| Relationship quality actual (positive) | -.78 | **<.001** | [-1.02;-0.53] |
| Coresidence (yes) | -.51 | **<.001** | [-0.76;-0.26] |
| Informal help wish (yes)^g^ | .51 | **<.001** | [0.26;0.76] |
| Benefits^h^ | -.02 | **<.001** | [-0.03;-0.01] |

*Note*. *N*=958.

^a^Standardized regression coefficient

^b^*p*<.05 printed in bold letters

^c^Adjustment variables related only to caregivers

^d^Dichotomous variable: female=0, male=1

^e^Final regression model: corrected *R*^2^=.24, *F*(8, 948)=37.72, *p*<.001; 6 steps

^f^Measured with the BSFC-s, range 0-30

^g^Dichotomous variable: CG would like to have more support in caregiving from friends or family

^h^Measured with the BBCS, range 0 – 56
